# Supporting the development of critical data literacies in higher education: building blocks for fair data cultures in society

**DOI:** 10.1186/s41239-020-00235-w

**Published:** 2020-11-24

**Authors:** Juliana Elisa Raffaghelli, Stefania Manca, Bonnie Stewart, Paul Prinsloo, Albert Sangrà

**Affiliations:** 1 Universitat Oberta de Catalunya-Estudis de Psicologia I Educació, Barcelona, Spain; 2 Institute for Educational Technologies-National Research Council of Italy, Genova, Italy; 3 Faculty of Education, University of Windsor, Windsor, Canada; 4 College of Economic and Management Sciences, University of South Africa, Pretoria, Republic of South Africa; 5 Estudis de Psicologia i Educació, Universitat Oberta de Catalunya, Barcelona, Spain

## Introduction

In the last ten years digitalized data have permeated our lives in a massive way. Beyond the internet ubiquity and cultural change outlined in what Castells (1996) called the network society, we are now witnessing a datafied society, where large amounts of digital data—the DNA of information—are driving new social practices. The most enthusiastic discourses on this abundance of data have emphasized the opportunity to generate new business models, with professional landscapes connected to data science and open practices in science and the public space (EMC Education Services 2015; Scott 2014). However, more recently, the rather naïve logic of data capture and its articulation through various algorithms as drivers of more economical and objective social practices have been the object of criticism and deconstruction (Kitchin 2014; Zuboff 2019). The university as an institution fell into this paradigm somehow abruptly, while striving to survive its crisis of credibility. The digitalization of processes and services was considered a form of innovation and laid the foundations for the later phenomenon of datafication (Williamson 2018). Initially, fervent discourses embraced data-driven practices as an opportunity to improve efficiency, objectivity, transparency and innovation (Daniel 2015; Siemens et al. 2013). The two main missions in higher education (HE)—teaching and research—went through several processes of digitalization that encompassed data-intensive practices. In teaching, the data about learning and learners collected on unprecedented scales gave rise to educational data mining and particularly to learning analytics (LA) (Siemens and Long 2011). While some argued about the value of learning analytics in informing teachers’ decision-making about pedagogical practices as well as learners’ self-regulation (Ferguson 2012; Roll and Winne 2015), research also uncovered naïve or even poor pedagogical assumptions on the power of algorithms to predict, support and address learning, which were connected to techno-determinist approaches to data (Ferguson 2019; Perrotta and Williamson 2018; Selwyn 2019). The studies in the field have pointed out how few connections there are between LA models and pedagogical theories (Knight et al. 2014; Nunn et al. 2016), the lack of evaluation in authentic contexts, the scant uptake by teachers and learners (Vuorikari et al. 2016a, b) and the social and ethical issues connected to the topic (Broughan and Prinsloo 2020; Slade and Prinsloo 2013; Prinsloo and Slade 2017). Moreover, the massive adoption of social media has crossed paths with learning management systems, creating new forms of data of which both teachers and students could be completely unaware (Manca et al. 2016).

In the aftermath of the HE pivot online and the resulting “pandemic pedagogies,” the problem of data usage and data ethics through the marketization of data and algorithms has emerged as a hidden consequence (Williamson et al. 2020). At the same time, the advancement of networked, open and pro-social research has increased data availability around the world (Bozkurt et al. 2020).

Judged against this complex framework, data literacy would appear to be an important skill to possess. Approaches such as that of D’Ignazio and Bhargava (2015) show the investigations made in education (in this case, adult education for civic participation) to generate agentic practices around datafication. Also, Pangrazio and Selwyn (2020) have investigated the ways HE and school students engage with personal data collected via social media and personal apps. Their design- and intervention-based research focused on improving understanding of the lack of transparency and monetization of data, but also uncovered passive attitudes among the students in the trade-off between data extraction and their usage of the digital environments surrounding them. In the case of HE, important reflections about the way students and teaching staff should engage with the academic and learning analytics systems yielded interesting considerations of the need for privacy by design, usability and engagement and transparency in students’ data usage (Jivet et al. 2020; Tsai and Gasevic 2017).

Analysis of data collection and visibility has highlighted another side of data practices, entailing positive connotations in contrast with the prior view of data usage as a form of surveillance. The paradigm of open science, which invited citizens to engage, explore and contribute to data collection processes in research, was deemed a powerful tool for innovating in science communication and a way of promoting informal learning (Owen et al. 2012). Moreover, scholarly practice might address new connections between research and teaching through the use of open data as open educational resources (Atenas et al. 2015), moving towards a widespread scientific culture. However, actual practices in HE reveal several issues regarding the implementation of these types of innovation (Raffaghelli 2018).

Based on the above, the reader might grasp the problem of a fragmented phenomenology relating to data epistemologies and the required literacies (Milan and van der Velden 2016). Unquestionably, there is an increasing number of research projects and studies in social sciences that address a critical perspective on the problem of data practices in general and in HE as one of the key institutions of our contemporary society. Discussion about the literacies required is also becoming a clear matter of concern. But the ways in which society and scholars characterize data practices vary considerably, and are based on different “data epistemologies” (in a continuum from positive and proactive to negative and reactive) that contextualize the various discourses.

However, the lack of awareness of the fragmentation in the phenomenology of data practices prevents educators and higher education institutions (HEI) from intervening to set policies or implement a professional praxis beyond a limited, externally driven focus on data instead of a contextualized vision of data. It is worth considering the concept of “data culture” at this point. A data culture is seen as a situated, collective expression which encompasses professional identities, policies and specific practices relating to data, as part of an institutional culture. As such, the awareness that actors (learners, the professoriate in both its teaching and research activities, staff, HEI management and even families supporting the students) have of the contextual and material characteristics of data imaginaries could potentially provide the basis for uncovering power issues, misrepresentation and inequities, and thereby pave the way to building fairer data practices. We must not forget that HE has been characterized by its commitment to advancing knowledge in society and, more recently, to promoting the development of capacities to thrive as creative and responsible citizens (Fikkema 2016; McAleese et al. 2013). In the case of datafication and all it entails, with the advancement of artificial intelligence and the Internet of Things as marketable innovations, the complex tension between the goals of a neo-humanistic perspective and the requirements of the technocracy (which has been a matter of discussion since the beginning of the university) has become even clearer. However, it is also clear that the role of the university is to blend advanced, interdisciplinary theoretical reflection with empirical research and practice in the field of datafication within a space of meaning-making (particularly university teaching). In such a space, as envisioned early on by Humboldt, academics and students engage in a conversation which ultimately pushes the latter to take an active part in addressing the problem of data practices and cultures as reflective citizens and professionals (Pritchard 2004). On these basis, the university is called on to mediate meaning-making through activities such as collaboratories, workshops, professional development and quality evaluation exercises in addition to actual research activities. These are spaces that ensure that the conceptualization and problematization of datafication are kept at the forefront of the agenda both within and beyond the university. Moreover, curriculum design, with its frameworks of competence promoting active and engaging pedagogical practices, acts as a sort of circle of positive reification of knowledge and entails intense reflection over the existing knowledge (and concepts) of datafication and data practices.

The main goal of this Special Section is to advance the discussion on data practices in HE towards constructing an agenda of critical reflection regarding the literacies required. We anticipated that the EdTech community may react in unexpected ways to the questions proposed in the call, which were intended to act merely as ice-breakers. Nonetheless, the empirical papers received could have been started one or two years before the submission of the final paper sent to our call.

Fairly predictably, a substantial group of contributions dealt with mainstreaming learning analytics and data literacy as a technical endeavour in HE. The studies finally included in this collection mostly focus on learning analytics as a way of informing pedagogical practices, and contain a certain degree of critical analysis of the design and deployment of such educational technology innovations.

As co-editors, we felt that in order to underpin the four papers included in this section as selected pieces we needed to present the puzzle of different perspectives on how HE contributes to the development of critical data literacies (Markham 2018; Tygel and Kirsch 2016) as a means of building fair data cultures. We decided that the presentation of this puzzle could take the form of a position paper outlining the steps we intended to take to address the complex phenomenology of data cultures and practices in HE. This task was based on the four areas of research in which the co-editors are involved, thereby providing a bigger, albeit incomplete, picture of how the four research papers selected could fit in. The order given to the critical perspectives described is based on the approach adopted to data practices observation, namely, moving from institutional strategies to professional skills to students’ literacies, and finally moving beyond the HE context. Although the four perspectives, together with the four selected articles, might not be entirely aligned in their critique approach to data epistemologies, they all converge in requesting a review and analysis of the social and educational impact of current data practices in HE.

Albert Sangrà provides the first perspective, having worked for over two decades in addressing the quality of online education in HE. Embracing a proactive data epistemology, he highlights the opportunity provided by data-driven approaches to analysing educational quality. At the same time, he unveils the criticalities of metrics and their meaning for the reputation of HEIs, disentangling the impacts of such instruments on both institutional culture and academics’ and students’ decisions and priorities. The second perspective, based on nearly ten years of research into the ethical concerns surrounding learning analytics, is that of Paul Prinsloo, who explores in depth the problems of students’ data and the usage of these data to produce a techno-structure for learning analytics. Prinsloo explores the problem through the conceptual lens of vulnerability as an inherent condition of students in the system. The third perspective, introduced by Bonnie Stewart, whose work also has a long tradition in the issues of professional digital identity and digital scholarship, builds on the need to construct critical data literacies to navigate data within the university and beyond, and the connected requirements of faculty development to achieve this goal. The fourth perspective, offered by Stefania Manca on the basis of her expertise in the field of informal learning and professional development through social media, relates to data usage “on the wildness” of social media beyond the university context. Her perspective embeds critical data literacies within social media literacy.

While there is no “one-to-one” relationship between the selected papers and these perspectives, the former sampled the need for data literacy among university staff to produce a common vision of quality in HE, taking into consideration the huge use of metrics in such an endeavour (Yang and Li 2020); they also addressed the complexities of privacy and data usage in the design of learning analytics (Cerro Martínez et al. 2020) and the criticalities of extracting text as data to characterize and analyse polemic constructs such as gender issues in students’ evaluation of practice (Okoye et al. 2020); and finally, they also explored a conceptual model for addressing educators’ data literacy to enable them to engage with teaching analytics through an informed and mindful approach (Ndukwe and Daniel 2020).

In the remainder of this paper, we will introduce the four perspectives followed by the contributions made by the four articles and proceed to discuss them. The conclusions draw on this rich synthesis of research work to build an idea of the literacies required to support the emerging fair data cultures in HE.

## Collecting data for success? Issues in using university rankings and the particular situation of online learning

Student success is a critical topic when analysing the quality of education. Aside from the discussion on what success could really mean, there is certain consensus with the idea that teaching quality is a central factor to the success of students in HE, as there is a strong correlation between teacher quality and student learning (Gibson and Lang 2019). In a similar vein, in 2013 the European Commission established a High Level Group on the Modernisation of Higher Education to draft a report on this area, which concluded that the quality of teaching is crucial for ensuring that HE reaches the highest standards (McAleese et al. 2013).

However, collecting data for success or, from another perspective, obtaining data to assess teaching quality, poses a challenge (Matosas-López et al. 2019). The data used for assessing teaching quality do not usually refer to teaching performance, but rather reflect other aspects. There is a pernicious trend to use the data we have rather than looking for the data we really need, or not considering the special characteristics of the particular topic under investigation (Pozzi et al. 2019). Although there have been attempts to show how data from learning management systems can be used as an indicator of student engagement (Beer et al. 2010) or, more recently, to obtain indicators that have been intelligently created by integrating different existing data sources (Daraio and Bonaccorsi 2016), much further research is required on how data can be best used to improve HE teaching and increase student success (Martin et al. 2017).

One of these indicators is university rankings, used to indicate teaching quality and the data used to feed these indicators. Comparative studies show that most rankings are still one-dimensional (Moed 2019), and teaching is the least considered dimension and has fewer indicators, a fact which is detrimental to the research. A study by Hou and Jacob (2017) shows, after the analysis of three of the most influential university world rankings, how the indicators used are based on data that do not seem to explain the description of the indicator itself. For example, in the ARWU system the three indicators used to predict the position of an institution in the university ranking are (1) the papers published in Nature and Science journals; (2) the values of the Science and Social Sciences Citation Index; and (3) the number of members who won Nobel Prizes and Field Medals, with the latter being the main indicator for quality teaching. Meanwhile, in the case of the QS and THE rankings, the most influential indicators are the expert-based reputation indicators.

Goglio (2016) highlights the issue of the audience addressed by the rankings. She argues that although there are multiple audiences with different needs and interests that could be interested in the information provided by the rankings, these usually address a generic recipient. To avoid this, several rankings have evolved in an attempt to become more specialized, sometimes creating subdivisions of the same ranking and sometimes providing new specific rankings (by discipline, age of the universities, or specific topics such as impact or internationalization). Multidimensional rankings have arisen in recent years that aim to deal with this multiplicity of audiences.

From a more internal perspective, Soh (2017) points out some of the “sins” of university rankings, concluding that “problems in world university rankings have been conducted mostly at the verbal level with little substantiation of supporting statistics. It is argued that discussion on ranking issues needs statistical evidential support” (p. 104). This statement focuses on a very relevant problem behind the discussion: the capacity for understanding when talking about data, in other words, the need for data literacy among all the actors involved. While statistics professionals may have a thorough understanding of the topic, teachers do not. In order to improve teaching performance, it is crucial to understand data and the opportunities that they can offer for improving teaching.

Data for assessing teaching quality have to be spread at different levels: macro (rankings that provide comparisons between institutions), meso (institutional scorecards that foster better performance within the universities) and micro (indicators that could help teachers to identify strengths and weaknesses in their teaching performance and implement improvements). There is currently a trend for considering only the macro-level indicators as high-stake measures (Gibson and Lang 2019). However, the three levels must be analysed separately because they provide different approaches to using data from the systems. On the one hand, decision-makers at HEI should be aware of and assume the growing concern in this area, as they are required to motivate organizational adoption and cultural change in this field (Macfadyen and Dawson 2012). On the other hand, at the micro level, data which feed quality teaching indicators should be strongly considered by the teachers. Learning analytics will be mainstreamed into HEIs, although extending data literacy regarding data practices at the macro and micro levels across faculty will be an undeniable challenge. Data literacy has been argued to be a component of the teaching digital competence to be achieved by teachers (Risdale et al. 2015).

One of the more evident junctions where the shortcomings of university rankings and the need for data literacy meet each other is online education. Although it has long been neglected by university rankings, it is the perfect scenario for developing data-based improvements to teaching quality to ensure success: everything gets recorded, everything can be retrieved, everything can be analysed. The visibility and usefulness of online education is growing, especially in these times of pandemic when schools and universities are being put into lockdown.

Given that online education has always been—and still is—under suspicion (Sangrà et al. 2019), data analysis could become an interesting ally in the gathering of evidence of teaching performance quality in online environments. Moreover, online education could become a driver for retrieving data that better characterize the quality of any kind of education and which have been difficult to obtain up till now; that is, the interaction between teachers and students, and between students in collaborative settings. Although it has been hard to obtain data about this key element, we seem to be on a promising path in this respect.

Different techniques have been developed, since data analysis lets us delve deeper into the interaction activities students and teachers perform and provides us with relevant information for identifying those models of online education that are based on reciprocal feedback between actors. For example, Ammenwerth and Hackl (2017) carried out a system based on structural network analysis, providing an analysis of the intensity and direction of interaction in online learning settings. More recently, Ammenwerth et al. (2019) presented a set of indicators tailored to cooperative online-based learning environments, where interaction and cooperation are a means of fostering higher levels of learning. This is not only important because of the contribution data analysis can make to online education, but for the opportunity it offers to improve the ways in which different online learning models are implemented, since it shows the actual importance of the impact of a higher level of interaction on online learning.

This leads us to a key question that should drive further research: What are the right questions to ask in order to gather the appropriate and necessary data for improving the quality of online teaching? In addition to any relevant answers that this question may generate in future research, we should start by helping teachers become more data literate so that we can have better prepared actors capable of assuming such an important role in HE.

## Student vulnerability, agency and learning analytics: an elephant in the room?

Learning analytics aims to, inter alia, enhance our understanding of students’ learning (Gašević et al. 2015) and learning journeys and assist institutions in identifying students who are vulnerable and/or at risk of failing or dropping out (Parkes et al. 2020; Siemens and Long 2011). Student vulnerability has therefore been an integral part of the evolution of learning analytics in HE and has found specific expression in, for example, pondering the ethical implications of learning analytics (Slade and Prinsloo 2013; Prinsloo and Slade 2016). As learning analytics increasingly involves machine learning (ML), various forms of artificial intelligence (AI) and algorithmic decision-making systems (Prinsloo 2017), a critical re-evaluation of student vulnerability is of crucial importance for the current debate on data cultures in HE (Archer and Prinsloo 2020).

Vulnerability, in general, and especially student vulnerability in learning analytics can be considered “under-theorized” (Mackenzie et al. 2014; Prinsloo and Slade 2016). Being identified as vulnerable, in general, means membership of a sub-group and non-conformance to the criteria of “normal” or “non-vulnerable” or being “deficient” in some respects (Broughan and Prinsloo 2020). As such, vulnerability becomes a “label” (Luna 2009) and increasingly a permanent, digital part of an individual’s profile (e.g., Mayer-Schönberger 2009). The data and categories used to define student vulnerability are often “zombie categories” (Archer and Prinsloo 2020; Gullion 2018)—“categories from the past that we continue to use even though they have outlived their usefulness and even though they mask a different reality” (Plummer 2011, p. 195).

In the context of education, these labels become a voice-over layered on top of students’ learning (Slade et al. 2019) and accompany students for a particular course or semester, or even for the duration of the programme, and may follow them long after graduation. Aside from considering the permanence of such a label (and its implications—see Mayer-Schönberger 2009), there is a real danger that in an attempt to address students’ vulnerability, instead of improving it, the vulnerability may become pathogenic (Prinsloo and Slade 2016). It is therefore crucial to consider student vulnerability in the context of student agency (Jääskelä et al. 2020), as well as that found in the nexus of students’ habitus and agency, disciplinary and institutional contexts—efficiencies, responsiveness and resources, and macro-societal factors (Subotzky and Prinsloo 2011). While some of the discourses on student agency emphasize grit, persistence and a “can-do” attitude, they often forget that student agency is not only situated in a particular context and flow deriving from students’ habitus, but is, as such, a constrained agency (Subotzky and Prinsloo 2011) and is entangled in intergenerational structural arrangements and power (Strayhorn 2014).

In this brief reflection on the nexus of student vulnerability, agency and learning analytics, we have to proceed on the basis that learning analytics aims to mitigate student vulnerability and risk emerging from moral and contractual obligations to care (Prinsloo and Slade 2016; Slade and Prinsloo 2013). In doing so, learning analytics collects, measures and analyses student data and categorizes students according to institutional and researcher understanding (or lack thereof) of student agency and vulnerability. Student agency is entangled in and emerges from students’ habitus and dispositions (and their understandings and enactments of these), institutional and disciplinary habitus and dispositions and macro-societal changes and impacts (Subotzky and Prinsloo 2011). In light of the fact that vulnerability is under-theorized, we will now briefly map understandings of vulnerability before considering some points for further consideration when using learning analytics to identify and address student vulnerability.

Vulnerability is not a characteristic unknown to humans and is possibly our most defining (Mackenzie et al. 2014) and, in its essence, corporeal characteristic (Butler 2004, 2009). Acknowledging vulnerability as “an ontological condition of all human existence” (Mackenzie et al. 2014) does not, however, mean that we are all equally vulnerable. Judith Butler speaks of a “differential distribution of vulnerability” (Bell 2010, p. 147). Vulnerability is often seen as the result of a combination of characteristics, thereby defining “vulnerable as a fixed label on particular subpopulations” which “suggests a simplistic answer to a complicated problem” (Luna 2009, p. 124). Addressing individuals’ vulnerability may require “more than one answer” as “different types of vulnerabilities can overlap” (Luna 2009, p. 124). Luna (2009) therefore suggests that vulnerability is, per se, relational and layered. Butler, in her 2016 article “Rethinking vulnerability and resistance”, not only destabilizes the notion of vulnerability, but also expands its understanding as “a relation to a field of objects, forces, and passions that impinge upon or affect us in some way” (p. 16). If vulnerability is, as Butler (2016) suggests, relational, it follows that “vulnerability is not a subjective disposition” (p. 16) and is “neither fully passive nor fully active, but operating in a middle region” (p. 17).

Understanding vulnerability as layered is further enriched by Mackenzie et al. (2014) taxonomy of vulnerability, which comprises three distinct, but connected and often overlapping, sources of vulnerability, namely inherent, situational and pathogenic. Inherent vulnerability refers to humans’ intrinsic or corporeal vulnerability, which, depending on the situation context, may be mitigated or worsened, whether temporally, intermittently or permanently. Mackenzie et al. (2014) acknowledge that the inherent and situational categories are not “categorically distinct […] Both inherent and situational vulnerability may be dispositional or occurrent” (p. 8). Of particular importance to this reflection is Mackenzie et al. (2014) claim that “inherent and situational vulnerability give [sic] rise to specific moral and political obligations” (p. 8) not only in providing assistance, but in reducing the risks. The third type of vulnerability—pathogenic vulnerability—may paradoxically arise as a result of providing assistance.

Most of the work on student vulnerability and institutions’ obligations towards reaching out and supporting students with vulnerabilities are founded on the contractual (legal, social and moral) agreements between institutions and students (e.g., Prinsloo and Slade 2014, 2016; Slade and Prinsloo 2013). Butler’s (2012) discussion of the work of Emmanuel Levinas and Hannah Arendt destabilizes the contractual basis of the ethical obligations and proposes that the vulnerable person (student) “demands” a response. Butler (2012) states that our ethical obligations are, “strictly speaking, precontractual” (p. 140) even when we do not know the other, or choose the other. She further states that “reciprocity cannot be the basis of ethics, since ethics is not a bargain” (p. 140). By its very nature, the unsolicited demand arising from another’s vulnerability renders, in the case of learning analytics, the lecturer and support and administrative staff representing the institution vulnerable. The fact that in identifying vulnerable students, institutions themselves become vulnerable resembles the research by Prinsloo and Slade (2017). But, while Prinsloo and Slade (2017) would have claimed that the institution’s vulnerability came as a result of its contractual obligations towards students, to not only be responsive but also response-able, the work of Butler (2012) provides a different foundation to the contractual one, namely the duty of care that comes into being as a result of sharing a space, a learning journey. Lastly, we also have to consider Butler’s (2016) notion that vulnerability and agency are not, necessarily, opposites, or that vulnerability excludes agency, or that agency excludes vulnerability. Butler (2016) proposes that vulnerability can be a basis for resisting the conditions from where the (intersecting) vulnerabilities arise.

To summarize, while all students share a vulnerability due to their humanness (Butler 2004, 2009), not all students are equally vulnerable, and students’ vulnerabilities are layered and dynamic, ameliorated or worsened in relation to a field comprising other actors, human and non-human, and other layers. The data we have, notwithstanding its increasing granularity, immediacy, variedness and detail, are to a large extent nothing but proxies and time-stamped snapshots of an individual’s agency in a particular context and time and in relation to a broader field, in response to or as a result of intersecting layers of differential vulnerability.

So whereto from here? Luna (2019) identifies some very useful steps towards moving away from seeing vulnerability as an inherent characteristic of an individual and/or group and towards seeing it as a set of layers resulting in a state of vulnerability. The first step, according to Luna (2019), is to identify the different intersecting layers. It is crucial to also map how the different layers resulting in more or less vulnerability play out in a particular context and what stimuli trigger the layers to assume different positions of permanence/importance. It is also important to explore and disentangle the “cascading of layers”—how the layers interact and how one specific aspect of vulnerability may trigger a cascading of pathogenic vulnerabilities in a particular context (Luna 2019). The second step is to rank the different layers resulting in vulnerability with regard to their harmfulness in a particular context. Of particular importance would be identifying those layers resulting in vulnerability that are cascading or that have a domino effect. These layers have a “differential strength and damaging power” and “we should consider the dispositional structure of layers of vulnerability and assess what stimulus conditions can trigger them (their presence and probability of developing). Stimulus conditions relate to layers with the context, with the actual situation and possibility of occurrence” (Luna 2019, p. 92). Of particular importance is Luna’s (2019) proposal that three kinds of obligations can be applied to and arise from the previous ranking of layers and to the identification of the various stimulus conditions. The first obligation is “not to worsen the person’s or group’s situation of vulnerability (be this with a protocol intervention or with a public policy). Thus, we should avoid exacerbating layers of vulnerability” (p. 93). The second obligation focuses on the eradication of layers of vulnerability. In cases where a particular layer of vulnerability cannot be eradicated, we should attempt to minimize the impact of these layers. “Finally, these obligations can be expressed through different strategies such as protections, safeguards, as well as empowerment and the generation of autonomy” (Luna 2019, p. 93).

In conclusion, learning analytics has evolved into an established research focus and practice and there is increasing evidence of how the collection, measurement and analysis of student data changes our understanding of learning, student retention, our pedagogies and the support needed by students. Integral to the aim of learning analytics is identifying students who may be vulnerable or at risk and to assist them in realizing their own agency with the support of the institution. The appropriateness and effectiveness of mitigating student vulnerability and supporting their agency lies in having a critical, nuanced understanding of the layered-ness and relational nature of student vulnerability.

## Complicated solutions to complex problems: addressing educators’ data literacy

As digital platforms increasingly become sites for education (Perrotta and Williamson 2018), particularly in the midst of the unprecedented if haphazard #PivotOnline sparked by COVID-19, the reality that educational technologies are sites for data extraction needs to be recognized and navigated by learners, educators and decision-makers. As explained here, data practices and the relative techno-structure (the choices made about digital platforms and the way data is extracted) are embedded into HE institutional culture, taking the form of a data culture. In order to navigate this new reality effectively, we need to reconfigure and bring into focus our societal understandings of what it means to be literate in regard to data.

The concept of literacy goes beyond the decoding of text to making a meaning out of new and emergent digital modalities. The idea of “new literacies” (Cope and Kalantzis 2000; Lankshear and Knobel 2004) emerged through the 1990s and the first decade of the twenty-first century as a response to globalization as well as to digitization. The New Literacies framework asserted the need for literate citizens to be able to navigate the pluralism of contemporary culture as well as the presence of digital tools in society. New literacies are not simply technical skills; rather, New Literacies theorists distinguish between the use of digital technologies for what they call “new technical stuff” and “new ethos stuff” (Lankshear and Knobel 2007). They assert that the “technical stuff”—or the moving beyond analogue and typographic means of sound, image and text production to digital forms—is less central to new literacies than the participatory ethos and practices made possible by the Web 2.0 infrastructure of the internet. These new ethos practices emphasize “mass participation, distributed expertise, valid and rewardable roles for all who pitch in” (Lankshear and Knobel 2007, p. 18), whether or not digital technologies are utilized.

The more a literacy practice privileges participation over publishing, distributed expertise over centralized expertise, collective intelligence over individual possessive intelligence, collaboration over individuated authorship, dispersion over scarcity, sharing over ownership, experimentation over “normalization,” innovation and evolution over stability and fixity, creative-innovative rule breaking over generic purity and policing, relationship over information broadcast, and so on, the more we should regard it as a “new” literacy. (Lankshear and Knobel 2007, p. 21).

More broadly, Hobbs (2008) overviews four different approaches to new literacies, but notes that all share an emphasis on the constructed nature of audiences and authorship, the circulation of messages, and meanings, and the exploration of texts as representative of social realities and ideologies. These general tenets of new literacy have shaped digital literacies education for over a decade, encouraging reflection and hands-on experimentation. Within these new literacies frameworks, developing the practices and literacies that enable full participation and meaning-making has often been framed as an immersive form of literacy (Savin-Baden et al. 2010) in which understanding is experiential. Gee (2012) emphasizes that new literacies are often learned through situated practice and participation, with peers, in affinity spaces and informal learning contexts. Open and digital practice among educators is also often scaffolded in the same informal ways (Stewart 2018). But these approaches, with their focus on participatory and critical engagement in online spaces, pre-date the extractive nature (Erickson 2018) of contemporary datafied platforms, and thus are not designed to take into account the emergent data risks that learners are exposed to in immersive practice.

The datafication of educational spaces thus creates a gap in new literacies literature, and points to a need to expand what it means to be literate in the tools and ethos of contemporary meaning-making practice. Educators across K-12 and HE face a common learning curve at this moment: the systems we rely on for scholarship and education are increasingly designed to translate digital experience into behavioural data as part of the surveillance capitalism economy (Zuboff 2019). Race and gender biases built into algorithmic decision-making are also increasingly evident (Noble 2018), yet a systemic review of studies on educators’ data literacies (Raffaghelli and Stewart 2020) found that the vast majority address data literacy from a technical skill and data management perspective, rather than from any consideration of data as an emergent factor in the critical and social practices that make up the “new ethos stuff” of new literacies.

In an era of “smart” surveillant devices and platforms, questions of how educators make sense of datafication are urgent, yet research into the full complexity of educators’ data literacies and practices remains minimal. It is imperative, therefore, that our cultural and educational concept of data literacies be expanded to include the complex “new ethos stuff” concepts that guided the idea of new literacies, and that the new literacies literature continue to evolve in the face of datafication.

## Social media as socio-technical-cultural systems and implications for critical digital literacy

The complex data cultures in HE entail not only the usage of institutionalized platforms but also cover several intersections with social media. Students and university teachers move seamlessly from one side to the other of the digital ecosystem, generating several forms of tension for the data that can be captured (by the institution) and the data that escape into the wilderness of social media, feeding external interests. The main actors are often unaware of this fact. It is worth noticing that educational research about social media has grown significantly in the last few decades and has focused broadly on social media and their use per se (Galvin and Greenhow 2020; Greenhow and Askari 2017; Rodríguez-Hoyos et al. 2015) or on specific social media platforms (Manca 2020; Manca and Ranieri 2016; Pimmer and Rambe 2018; Tang and Hew 2017). However, despite increasing research documentation about social media use for teaching and learning, studies on social media literacy (as opposed to media literacy) are still rare. Many scholars have stressed the importance of developing social media literacy and of considering diversity in the experience and values relating to social digital platforms (e.g., Van Den Beemt et al. 2020).

The manipulation of user behaviour via algorithms and the risks of challenging online civic engagement on social media platforms where misinformation is pushed in disparate ways has led scholarly research to advocate the development of social media skills for both students and teachers (Damico and Krutka 2020; Journell 2019; McCosker 2017). In this scenario, teaching and learning *about* social media has emerged as a specific research focus that demands renewed attention. Scholars have identified several areas of concern that present significant challenges for educators and civil society, along with curricular possibilities for teaching and learning about social media platforms. These include user agreements and use of data; algorithms of oppression, echo and extremism; distraction, user choice and facilitating access for non-users; harassment and cyberbullying; and gatekeeping for accurate information (Krutka et al. 2019).

Teaching and learning social media skills is particularly relevant when we consider that social media literacies encompass social and ethical aspects and not just technical skills alone (Pangrazio and Selwyn 2018). In this vein, social media literacy may be conceived as a combination of technological, cognitive, social and ethical skills needed for the critical evaluation (and use) of social media (Hobbs 2010; McDougall et al. 2015).

Despite general consensus on the importance of developing social media literacy, a current tendency is to employ general frameworks that address global and decontextualized digital skills or competencies for media and digital education. Two prime examples are the UNESCO Media and Information Literacy Curriculum for Teachers (also known as MIL), which combines educational goals regarding digital media studies with information literacy (UNESCO 2011), or the Digital Competence Framework (also known as DigComp 2.0) produced by the European Commission (Ferrari 2013; Vuorikari et al. 2016a, b). However, neither of these general frameworks, nor others like them, consider that using social media effectively demands the development of general digital literacy skills as well as the mastery of context-dependent practices (Dron 2019; Manca et al. 2021). These considerations are grounded in the nature of social media platforms themselves, which are considered as socio-technical systems whose information (software) level is closely intertwined with individual and societal levels: the interlinkages between technology, people and the social environments in which they are used is what characterizes these digital platforms (Krutka et al. 2019). In this vein, a combined perspective for investigating social media literacies that considers social media skills as a combination of global skills (transversal across different social media) and local skills (pertaining to a specific social media platform) needs to be intertwined with an examination of practices that may be decontextualized or situated and context-dependent (Manca et al. 2020).

Among the broad sets of skills associated with social media literacies, at least three critical issues deserve specific consideration in the context of this Special Section.

The first is related to the massive use of big data in social media research. While use of big data is an uncommon issue in educational contexts of limited scope, big data analytics has recently emerged as an important research area due to the pervasiveness of Web 2.0 technologies. Use of big data for learning purposes poses a number of important challenges to students and educators. Data quality, velocity, data availability and natural language processing techniques are only some of the many technical challenges that remain to be addressed (Ghani et al. 2019). Computational big data methods and mixed-method social network analysis (MMSNA), which may be employed to complement the measurement of user engagement on a social media platform (Froehlich et al. 2019), requires specialized technological and cognitive skills in order to retrieve massive datasets and apply sound interpretative approaches to the data (Manca et al. 2016). Moreover, limitations in collecting methods can have consequences in terms of the representativeness of the data due to restrictions on the number of calls to APIs per time unit and on the number of data items returned per call (e.g., the case of Twitter), and to the prohibition of using automated scripts to collect user data (e.g., Facebook). Today, only a certain (ever-smaller) number of academic researchers have access to server-level data (Olmstead and Barthel 2015).

The second concern regards the topic of ethics in social media research. The exploitation of users’ data for educational purposes and research aims presents a number of controversial new issues. Today, there is no broad consensus on how to ethically treat data collected on social media, and scholars continue to debate their public or semi-public nature (Sloan and Quan-Haase 2018). For instance, obtaining informed consent from social media platforms such as Twitter, which is considered a broadcast medium, has been debated among academics, who assume different positions depending on “discipline, the level of understanding that ethics committees have about the nature of social media data and whether proposals using “scraped” data should be classified under primary collection or secondary analysis” (Sloan and Quan-Haase 2018, p. 670). In this line, scholars have not yet resolved the ethical issue of whether public tweets are by default public data (Kitchin 2014), and they caution against considering social media posts publicly available where user expectations of privacy are lacking (Franzke et al. 2020), In general, teachers and scholars need to be aware that “just because social media data are public, does not mean people do not have context-specific and data-specific expectations of privacy” (Gruzd et al. 2020, p. 1).

Finally, the third concern regards some indications from the interdisciplinary area of digital humanities. In the field of cultural studies, for instance, scholars have investigated the rapid transformation of data about historical events with the increasing usage of digital technologies in mediatization processes that result in digital memories (Garde-Hansen et al. 2009). The fluid relationship between social media technologies, cultural memory and forms of commemoration of an historical event, such as the Holocaust, on the internet has been reported as an example of the transcultural mediation process between history and memory, and between memory, technology and culture (Pfanzelter 2016). While the dynamic nature of Web 2.0 is now part of our daily socio-cultural practices, enabling the permanent addition, modification, deletion and reconstruction of private and public content thanks to social media platforms, for the purposes of this editorial it is important to remark that the utopian ideas of unlimited archiving in terms of time and space must be compared with the idea that “the sustainable archiving of digital data still depends on institutional preferences, hardware selection, file formats, software decisions and archiving practices. Beyond that, individual users determine how and what they do. However, this latter practice increasingly decides […] which parts of our cultural legacies will ultimately be archived and affect the indelibility of digital information” (Pfanzelter 2016, p. 223). These considerations have profound implications for how we conceive the creation, archiving, retrieval and reuse of data in terms of critical digital literacy applied to social media.

## Four selected works: the contribution of this Special Section

Advancing in the framework depicted by the four perspectives requires a process of progressive mapping and integration of data practices to construct a new analysis of the impact, institutional policies and conceptual and instrumental tools, and support teachers and students to engage with data in an agentic way. The contribution of this Special Section lies in the reflections and empirical work made to instantiate the elements of data cultures in HE.

Exploring this area in greater depth, through their participatory design-based research, Cerro Martínez et al. (2020) highlight the crucial value of teachers and pedagogical experts’ engagement from the moment that learning analytics are conceived. Taking into consideration the relevance of asynchronous online discussion activities, but also the complexity entailed by them, the authors aim to leverage student awareness and participation in collaborative activities through the mediation of the DIANA analytics tool. The authors acknowledge the limitations of technology to support the complexities of a pedagogical activity, but their participatory design reveals the relevance of analytics *if* sense is made of the pedagogical representations (as a cultural aspect) prior to the data-driven practice.

Okoye et al. (2020) use analytics to disentangle gender bias in students’ evaluation of teaching. The authors claim that user-centric analyses are useful in both a reactive and a proactive data epistemology, in the sense that they consider the unprecedented scale of text-based data as an opportunity to reflect on the teacher-student experience, by developing the Educational Process and Data Mining (EPDM) model. However, they apply the model to uncover gender issues within the students’ evaluation of teaching, as a means of developing greater awareness on such critical issues in HE.

As for the study of Ndukwe and Daniel (2020), a systematic review of the literature is used to develop a reflection on the way teachers can appropriate the power of teaching analytics (TA). The authors insist on the need to connect the dots of teaching analytics, learning analytics and learning design, as forms of representation (usually embedding visual representations) to improve the quality of teaching. The authors set the review to establish a framework describing the various aspects of TA and to develop a model that can enable the readers to gain more insight into how TA can support the continuous improvement of teaching and learning. The authors adopted a tripartite model to carry out a comprehensive, systematic and critical analysis of the literature of TA within a period spanning from 2012 to 2019. The results of the study have led to the development of a conceptual framework for TA and established the boundaries between TA and LA. Indeed, they propose a Teaching Outcome Model (TOM) as a theoretical lens to guide teachers and researchers to engage with data relating to teaching activities, to improve the quality of teaching.

The work of Yang and Li (2020), the authors investigate the contribution of stakeholders’ data literacy to build a shared vision of data usages supporting the analysis of quality in HE. The authors highlight the fact that the majority of data in HE have not been transformed into actionable insights for quality enhancement, as the data practices are dispersed. They use the goal-modelling language iStar to present how stakeholders contribute to student success, and then discuss a competencies matrix of data literacy connected to such success. On this basis, the authors point to the complexity of interactions and dependencies among stakeholders for student success. Their study helps to raise stakeholder awareness of the importance of data literacy and the need to collaborate in exploiting the vast data available to facilitate student success.

Overall, two of the selected works investigate the conception of the techno-structure that enables data-driven practices, for example, the designing and testing of learning analytics aimed at capturing complex constructs such as collaboration (Cerro Martínez et al. 2020) or gender bias in students’ evaluation of teaching (Okoye et al. 2020). The other two works consider the actionability of analytics and the actors’ skills and knowledge of them to keep improving their practices. Specifically, Ndukwe and Daniel (2020) underline the impact of analytics as enablers of teachers’ reflection on the quality of their teaching, and Yang and Li (2020) explore the way HE stakeholders’ data literacy contributes to students’ educational success in a holistic way.

While these four contributions are not conclusive and do not cover the full range of topics expressed in the four perspectives addressed by the guest editors, they converge in expressing the diversity of debates around data practices and data cultures in HE as well as the central role of academic and learning analytics in relation to other critical reflections such as data capture across the techno-structure. Particularly, the issue of media ecosystems when using social media, which has been a matter of reflection when considering lifelong learning strategies, has yet to be properly explored from the point of view of data literacy for/in HE.

## Conclusions

Through the contributions made to this Special Section, both in the form of original articles and in the effort made by the editors to synthesize their research-based perspectives, the dots begin to be connected. This is not to say that there are not leaps to be made from one position to another; however, in order to outline future areas of research and practice, we summarize below some of the main issues arising in the contributions.

The definition of data practices given was purposely broad, spanning from educational data mining to open research data for teaching as part of responsible research and innovation. As the goal of the call for papers was to provide not only a description of ongoing data practices but also a critique of their limitations, the weight and positive consideration given to technological developments across many of the contributions should not be overlooked. The empirical research on learning analytics and the discussion over university rankings raised the idea of data-driven practices as a valuable source of development which needs to be furthered in order to achieve precision and effectiveness. In the same vein, the idea of data literacy is developed through the literature review and the goal-oriented analysis built on the conviction that becoming familiar with current data practices and their techno-structure will enable participants to engage fairly within the HE data culture. Where there is clear tension between the proactive vision of data practices and the critique of them, we then find a discussion on students’ vulnerability in relation to the capture, elaboration and usage of their data. In connection to this position, we could also place the perspectives on the need to unpack data literacy as a complex set of abilities which include the ability to read the political and ethical consequences of datafication across platforms and particularly on social media. None of the contributions engaged with the potentials and pitfalls of open data for learning and the transparency of educational research, thereby illustrating the fragmentation in data practice discourse, which is also embedded within a same institutional data culture.

Despite these nuances in the positions adopted by the authors and editors on data cultures in HE, there were strong areas of convergence. These regarded the compelling need to render visible the invisible, namely, the social and cultural structure motivating data production, elaboration and usage. Such transparency could be achieved in different ways, for example, by uncovering the students’ vulnerability in the techno-structure, as actors at the base of a hierarchy, regardless of the horizontal model the university claims to embrace; by participating in the design of data practices to understand and discuss pedagogical and organizational interests and evaluate the impact of data-driven operations; by putting further emphasis on assessing the social impact of data usages, in terms of the expected performances and the idea of quality pursued by the institution; and last, but not least, by developing a complex set of skills that encompass the literacies required not only to read or understand data *as text,* but to take an active part in the creative and participatory processes which use data as just a *mediational artefact.*

All the contributions in this Special Section highlighted the relevance of disentangling the materialities of data practices. In this endeavour, data become a mediational artefact, a conceptual, situated and multi-layered object which promotes activity and reflection between the actors within the cultural and social structure they are engaged in.

All in all, each university should build spaces for their actors (students and staff) to engage in actions that lead to awareness as well as concrete actions and research in the following areas:

Identifying problems in the data structures and practices**.**

- Focusing on a problem of the educational context of practice, understanding the learner role within the problem, involving stakeholders, discussing privacy, algorithms, commodification and technological usages in general.
- Considering how solutions to an educational problem can be informed and created through an ecological use of data.

Exploring data usages:

- Identifying all types of data sources, including open research and government data.
- Exploring data and their purposes, properties and quality; understanding how data is generated; understanding how data can be extracted; using multiple (quan-qual) measures/sources of data; understanding how to analyse, manage and aggregate data, enacting a collaborative use of data within the professional activity.
- Exploring sources of data, particularly open data, and embracing data activism by breaking the silos of data production (government, companies, from research data to data in education).

Transforming data into information.

- Understanding how data can be visualized, represented and shared; generating hypothetical connections to instruction; testing assumptions; assessing patterns and trends; synthesizing diverse data; articulating inferences; summarizing and explaining data.
- Considering the ethical concerns of all data-driven processes and the ways in which statistical synthesis captures/pays attention to a phenomenon.

Evaluating outcomes.

- Determining the next pedagogical practices, monitoring learners’ reactions and engagement with data, diagnosing additional student needs, making adjustments, understanding the limitations imposed by decision-making contexts.
- Supporting learners’ critical data literacy and pedagogical data literacy by discussing the data assemblages adopted throughout a learning process/activity.

This work certainly requires what Selwyn and Gašević (2020) instantiated in their contribution: a continuous interdisciplinary conversation to come to terms or, even better, to generate approaches which are aware of the agendas in education/social sciences and computer science. As pointed out by Gašević (in conversation with Selwyn), there is careless use of learning analytics by some HE managers and reductive discourses mainly when introducing such data-driven approaches to complex, unsolved problems in HE, for example, student dropout. However, computer scientists are not unaware of the perils of reductionism and are trained to understand that their developments are based on incomplete and synthetic representations of the reality. Moreover, computer scientists require domain experts in order to overcome initial design problems relating to ill-defined problems, as in the case of educational processes.

However, with the current state of affairs, it is not unusual to see “techno-solutionism” around problems that have been a sort of managerial nightmare. The forms of de-responsibilization which the objectivist positionings around data practices create is a clear expression of the pressure on the system to produce results fast. The faster, the simpler, the better, which entails less attention (as claimed by Selwyn and Gašević) to the need for complex interventions where data-driven practices are just another piece of the puzzle. The ethics, the politics and even the narratives and their aesthetics are embedded in the materiality of data, as the result of an extremely laborious social and semiotic elaboration (Whitman 2020). Moreover, good data visualization and actionable representations never come from simple, direct data collection, as manifested in all the contributions in this Special Section and reinforced by Whitman (2020) and Selwyn and Gašević (2020). Another crucial endeavour will be to embrace a post-colonial and post-feminist approach in analysing the techno-structure and building a data culture. As Prinsloo (2020) pointed out in relation to vulnerability here but also with the problem of “data frontiers” elsewhere, the data used to represent collectives or to provide services to them must not cause harm. In this regard, the deeper vulnerabilities acknowledged for some of those collectives should become a driving force for deconstructing algorithms and their conceptual basis. It is not a question of automatizing inequalities, to paraphrase Eubanks (2018), but a matter of revisiting the same principles of services and pedagogical practices in HE.

To conclude, since the sources of data are extremely molecular against the complex, incommensurable and ephemeral symbolic representation we want to turn them into, engaging in such an endeavour would leave deep impressions and trigger insights of heuristic value for identity as well as cultural construction. Nonetheless, such heuristic value is central to the university’s dialogue with society.
